# Extracellular water may increase with age and be independently and negatively associated with muscle strength and physical function in older adults: A cross‐sectional study

**DOI:** 10.14814/phy2.70931

**Published:** 2026-06-10

**Authors:** Yujiro Asano, Tsukasa Yoshida, Kenji Tsunoda, Keiichi Yokoyama, Yuya Watanabe, Yasuko Yoshinaka, Tomohiro Okura, Misaka Kimura, Yosuke Yamada

**Affiliations:** ^1^ Laboratory of Sports and Health Sciences, Graduate School of Biomedical Engineering Tohoku University Sendai Japan; ^2^ Center for Physical Activity Research National Institutes of Biomedical Innovation, Health and Nutrition Settsu Osaka Japan; ^3^ Institute for Active Health Kyoto University of Advanced Science Kameoka Kyoto Japan; ^4^ Department of Medicine and Science in Sports and Exercise, Graduate School of Medicine Tohoku University Sendai Japan; ^5^ Institute of Health and Sport Sciences University of Tsukuba Tsukuba Ibaraki Japan; ^6^ Non‐profit Organization Genki‐Up AGE Project Kameoka Kyoto Japan; ^7^ Faculty of Sport Study Biwako Seikei Sport College Otsu Shiga Japan

**Keywords:** BIA, bioimpedance, BIS, community‐dwelling older adults, physical function

## Abstract

Whether extracellular water (ECW) increases with aging in humans and whether it is negatively associated with muscle function remains unclear. We aimed to clarify the associations of segmental intracellular water (ICW) and ECW with age and their independent associations with muscle strength and physical performance. This cross‐sectional analysis included 1102 older adults; segmental ECW and ICW in the thigh and arm were assessed by bioelectrical impedance spectroscopy (BIS). Knee extension and handgrip strength assessed muscle strength, and the timed‐up‐and‐go test, 6‐m maximum walk time, chair stand, vertical jump, and single‐leg balance tests assessed physical performance. Among older age groups, covariate‐adjusted thigh ECW was higher, whereas ICW and total body water were lower. Arm measures demonstrated similar age‐related patterns, although less consistently, with sex‐specific variation. Higher thigh and arm ECW levels were independently and negatively associated with muscle strength and physical performance in the corresponding segment (thigh: |β| = 0.12–0.31; arm: |β| = 0.12–0.20) after covariate adjustment. Conversely, higher arm and thigh ICW levels were positively associated with muscle strength and physical performance in the corresponding segment after adjustment. ICW may reflect functional muscle, whereas ECW may not be merely a passive non‐contractile compartment; rather, it may be a negative correlate of muscle strength and physical performance. Assessing segmental ECW alongside ICW may be essential to detect age‐related differences in muscle quality.

## 1 INTRODUCTION

Age‐related mobility decline, sarcopenia, and frailty increase disability risk, the need for long‐term care, and mortality, representing a significant burden on aging societies. Muscle mass and volume assessed by dual‐energy X‐ray absorptiometry (DXA), magnetic resonance imaging, and computed tomography are widely implemented to capture these changes; nonetheless, such quantity‐based indices do not consistently exhibit strong associations with activities of daily living, physical performance, or mortality risk (Cawthon et al., 2019; Evans et al., 2019, 2023; Newman et al., 2006; Schaap et al., 2013; Wang et al., 2020). Importantly, this inconsistency may be due to these imaging techniques measuring total muscle volume, including both contractile muscle fibers and non‐contractile components (Evans et al., 2019, 2023; Yamada, 2018).

Skeletal muscle contains a large water amount, which can be broadly divided into intracellular water (ICW), located within muscle cells, and extracellular water (ECW), which exists in the interstitial fluid and blood plasma. The former is considered a “functional muscle” indicator, as it reflects muscle cell volume and the amount of contractile apparatus (Lorenzo et al., 2019; Yamada, 2018; Yamada et al., 2010). ECW reflects extracellular fluid volume, which may partly capture expansion of the extracellular space related to edema, inflammation, fibrosis, and fat infiltration associated with aging and inactivity. These changes may occur alongside non‐contractile structural changes such as IMAT and ECM accumulation (Hooijmans et al., 2024; Pagano et al., 2024; Stock & Thompson, 2021; Yamada, 2018). Accordingly, substantial research using different approaches has reported that indices reflecting reduced relative contractile muscle content are associated with lower muscle strength, poorer physical performance, and higher mortality (Addison et al., 2014; Asano et al., 2023; Asano, Tsunoda, et al., 2025; Delmonico et al., 2009; Iwasaka et al., 2023, 2024; Kitagawa et al., 2023; Kuschel et al., 2022; Yamada et al., 2017). Specifically, imaging and histological studies have focused on structural changes such as increased IMAT, whereas bioimpedance‐based studies have examined fluid‐related indices such as a higher ECW/ICW ratio (Prado et al., 2025). Nevertheless, these composite indices do not allow for the disentangling of the extent to which these adverse outcomes are driven by increases in ECW rather than decreases in ICW.

In animal models, ECW, ECM, and IMAT expansions have consistently affected muscle force and power reductions, even when muscle fiber size is preserved, likely through impaired force transmission and altered mechanical properties of the muscle–extracellular matrix complex (Biltz et al., 2020; Zhang & Gao, 2014). Therefore, beyond muscle fiber atrophy itself, increases in extracellular water, connective tissue, and fat may be independently associated with lower muscle function. Nonetheless, in humans, whether ECW is merely a neutral compartment that does not contribute to muscle contraction, or whether it is negatively associated with muscle strength and physical performance (for example, by mechanically or metabolically impairing contractile function), remains unclear. If ECW simply reflects a fluid compartment that does not contribute to force generation, quantifying contractile tissue alone might be sufficient from a clinical standpoint. Conversely, if ECW increases with age and is independently and negatively associated with muscle strength and physical performance, quantifying non‐contractile components and fluid‐related indices such as ECW itself, rather than only “functional muscle mass” or the relative loss of contractile tissue, may provide additional insight into age‐related changes in muscle composition.

Regarding water compartments, numerous human studies have reported that ICW declines with age, reflecting reductions in muscle cell volume and the contractile apparatus, that is, a loss of “functional muscle cells” (Lorenzo et al., 2019; Yamada, 2018; Yamada et al., 2010). Conversely, cadaver and imaging studies have repeatedly demonstrated age‐related increases in extracellular components such as ECM and intramuscular fat (Yamada, 2018), which may be accompanied by ECW increases. Nevertheless, findings from studies directly assessing ECW in humans are inconsistent: some report age‐related increases in ECW, whereas others suggest that it is relatively preserved (Ohashi et al., 2018; Silva et al., 2005, 2007; Yamada et al., 2010, 2014); therefore, no clear consensus has been reached. This inconsistency may be attributable to differences in whether water is assessed at the whole‐body or limb level, the measurement techniques used and their accuracy, as well as sample size and study design. Additionally, most previous studies have relied on composite indices such as the ECW/ICW ratio, which do not allow disentangling the independent contributions of ECW and ICW. Consequently, it remains unclear whether functional decline is primarily driven by ECW expansion, ICW reduction, or both.

Here, we addressed these gaps by examining a large cohort of community‐dwelling older adults. We estimated ECW and ICW in the upper and lower limbs using bioelectrical impedance spectroscopy (BIS), currently considered among the most appropriate methods for segmental water assessment, as dilution techniques can only be applied at the whole‐body level, and multifrequency bioelectrical impedance analysis demonstrates limited accuracy for segmental measurements. In this study, we focused on segment‐specific measures, particularly in the arm and thigh, because these regions may better reflect muscle‐related water distribution and may be less influenced by non‐muscular tissues and organs than whole‐body measures. Using these segmental ECW and ICW estimates, we aimed to (1) describe age‐related differences in limb ECW and ICW and (2) examine, in particular, whether ECW is independently and negatively associated with muscle strength and physical performance after accounting for ICW and conventional covariates. We hypothesized that ECW, which tends to be higher in older individuals, would be negatively associated, whereas ICW, which tends to be lower in older individuals, would be positively associated with muscle strength and physical performance.

## 2 METHODS

### 2.1 Participants

Data were obtained from the Kyoto–Kameoka Study (Watanabe et al., 2020; Yamada, Nanri, et al., 2017), a cohort study of individuals aged 65–90 years. A baseline survey on daily living needs was distributed on July 29, 2011, to 16,474 community‐dwelling older adults without long‐term care certification; 12,054 (73.2%) returned valid questionnaires. Subsequently, we randomly selected 10 of 21 regions for health examinations and sent invitations to 4831 individuals, of whom 1379 (28.5%) participated from March to April 2012. After excluding (1) nine individuals who had received long‐term care certification before this study, (2) 94 individuals who did not measure or had incomplete BIS data, and (3) 174 individuals with incorrect covariate data, 1102 participants remained for the final analysis. This study was approved by the Ethics Committees of the Kyoto Prefectural University of Medicine (No. RBMR‐E‐363), Kyoto University of Advanced Science (No. 20‐1), and the National Institute of Health and Nutrition (No. NIHN187‐3 and 25M22) in accordance with the Declaration of Helsinki.

### 2.2 Body composition measurement

Using BIS according to the principles described in previous publications (Khalil et al., 2014; Lorenzo et al., 1997; Sato et al., 2020; Yamada et al., 2010), we assessed cellular water status using an SFB7 device (ImpediMed, Pinkenba, QLD, Australia). At the beginning of each measurement day, the system was calibrated with precision resistors supplied by the manufacturer to ensure data quality, and measurements were performed by previously trained operators. To ensure the reliability and accuracy of BIS data under field conditions, measurements were conducted using a standardized device setup and body position protocol. Food and beverage intake and medication use before testing were not controlled, and assessments were performed during both morning and afternoon sessions. Participants rested in a supine position for at least 15 min in a temperature‐controlled environment to allow for fluid stabilization and to minimize short‐term variability in hydration status.

Impedance data for the thigh segments were collected using the following methods. For thigh measurements, injection electrodes were placed on each side of the dorsal foot near the second and third metatarsophalangeal joints, and sensing electrodes were placed on both sides of the joint space between the femoral and tibial condyles. Regarding arm measurements, injecting electrodes were placed on each side of the dorsal hand near the second and third metacarpophalangeal joints, and sensing electrodes were positioned on both sides at the joint gap of the radial and ulnar styloid processes. The segmental length (L) was defined as the length of the bilateral segment and was calculated by doubling the right‐side segment length measured between standard anatomical landmarks. Specifically, measurements were taken from the femoral‐tibial joint cleft to the anterior superior iliac spine for the thigh and from the radial–ulnar styloid process to the elbow and from the elbow to the acromion for the arm.

Subsequently, bioimpedance data were modeled using the Cole‐Cole approach, enabling the calculation of ECW resistance (R₀) and total water resistance (R_∞_) across the thigh and arm under specified analysis parameters (5–500 kHz, 0% rejection). Next, the ICW resistance (R_i_) was obtained using the formula R_i_ = 1/([1/R_∞_] – [1/R₀]). The amounts of each segmental ECW and ICW were then calculated based on the equations ECW = (ρECW × L^2^)/R_0_ and ICW = (ρICW × L^2^)/R_i_, where ρECW and ρICW represent the resistivity constants for extracellular (47 Ωcm) and intracellular (273.9 Ωcm) water, respectively (Kaysen et al., 2005; Khalil et al., 2014; Lorenzo et al., 1997). Finally, total body water (TBW) volume in each segment was determined by adding the calculated ECW and ICW. The validity of BIS‐derived estimates of TBW, ICW, and ECW has been demonstrated previously relative to reference dilution methods (Kaysen et al., 2005; Lorenzo et al., 1997; Shiose et al., 2020). Moreover, between‐day reproducibility of the laboratory measurements was assessed; coefficients of variation and intraclass correlation coefficients were 2.0% and 0.969 for ECW, 3.4% and 0.896 for ICW, and 2.4% and 0.944 for TBW, respectively. Data exceeding the mean ± 3 standard deviations were excluded to minimize the effects of measurement error and outliers.

### 2.3 Muscle strength measurements

Muscle strength was assessed using handgrip strength (upper extremity) and knee extension strength (lower extremity). Handgrip strength was measured using a Smedley hand dynamometer (Grip‐D, TKK5401; Takei Scientific Instruments, Niigata, Japan). Participants performed two maximal‐effort trials for each hand with a short self‐paced rest interval while alternating sides, and standardized verbal encouragement was provided. The mean of the highest grip strength measurements from each hand was used. Knee extension strength was measured for each leg at a knee angle of 90°, with 0° representing full leg extension, while participants were seated on a dynamometer chair (TKK5710e; Takei Scientific Instruments Co., Ltd., Niigata, Japan). After standardized instructions and familiarization including practice trials, participants performed two maximal‐effort trials for each leg, separated by 1 min of rest, with standardized verbal encouragement. Participants were tested only after confirming adequate understanding of the task. The maximum strength value recorded for each leg was obtained, and the mean of these peak values was used. Detailed instructions for these measurements have been previously described (Kimura et al., 2012; Watanabe et al., 2020). Those judged unable to perform a valid maximal effort because of pain or difficulty understanding the instructions were not included.

### 2.4 Measurement of physical performance

Physical performance included the timed up and go (TUG) test, the 6‐m maximum walk time, the five‐times chair stand test (chair stand), the vertical jump index (VJI), and single‐leg balance with eyes open. The TUG test measures the time (s) required for participants to rise from a standard chair, walk 3 m at maximum speed, perform a turn, return to the chair, and immediately sit down without running. The 6‐m maximum walk time was recorded as the time required to walk the central 6‐m of a 10‐m walkway, excluding the first and last 2m, recording the time from the first attempt, and performing a second attempt if the first was unsuccessful. The chair stand test recorded the time required to complete five consecutive sit‐to‐stand movements on a stable, padded, and armless chair as quickly as possible. Vertical jump height was assessed using a Jump Meter‐MD (TKK5106; Takei Scientific Instruments, Niigata, Japan) after participants were familiarized with the procedure by performing two maximal jumps with lower‐limb countermovement: one allowing arm swing and the other requiring the hands to remain at the waist. The highest of the two jump heights was selected for subsequent analysis, and the VJI was calculated by multiplying the jump height by the individual's body weight (m × kg). Balance was assessed using one‐leg balance with eyes open, with participants maintaining an upright posture and hands on the waist, for a maximum of 120 s. Detailed instructions for these measurements have been described in previous studies (Kimura et al., 2012; Watanabe et al., 2020).

### 2.5 Statistical analyses

All statistical analyses were performed using R version 4.3.2 (R Foundation for Statistical Computing, Vienna, Austria), with statistical significance set at *p* < 0.05. We performed a power analysis using G*Power 3.1, assuming a correlation of *r* = 0.20, α = 0.05, and 80% power. This analysis indicated that the minimum required sample size was 191 participants. Our final analytic sample of 1102 participants exceeded this threshold. Participants with incomplete BIS or covariate data were excluded from the analytic sample. Therefore, the main analyses were conducted as complete‐case analyses. Histograms are provided in the Appendix S1 to aid interpretation of the distributions of the outcome and explanatory variables (Figure S1).

All analyses were performed separately by sex because body composition, fluid distribution, muscle strength, and physical performance differ substantially between males and females, and sex‐specific analyses were considered more appropriate for interpretation. First, an analysis of covariance (ANCOVA) was performed to compare the ECW, ICW, and TBW in the thigh and arm among four age categories (65–69, 70–74, 75–79, and ≥80 years), adjusting for height, weight, joint pain, status of alcohol consumption and smoking, history of chronic diseases, including hypertension, stroke, heart disease, diabetes, dyslipidemia, renal disease, prostate disease, cancer, osteoporosis, and other musculoskeletal disorders and Parkinson's disease, dietary intake of protein, fat, and carbohydrate assessed by the validated Food Frequency Questionnaire (Date et al., 2005; Imaeda et al., 2007; Tokudome et al., 2005), number of medications, and participation in sports/exercise and walking. Covariates were selected as common confounders considered relevant to both the age‐group comparisons and the regression analyses and were applied consistently across the adjusted models. Graphs were prepared using estimated marginal means and 95% confidence intervals, and linear trend tests for BIS variables across age categories were performed using polynomial contrasts. Additionally, percentage changes in BIS variables between consecutive age categories (e.g., from 65–69 to 70–74) were calculated.

Subsequently, multiple linear regression analyses were performed to examine the associations between ECW and ICW in the arm and thigh and upper and lower extremity muscle strength. Multiple linear regression was used under the assumption of linear associations, consistent with prior studies, to estimate the independent associations of ECW and ICW with continuous measures of muscle strength and physical performance. Two regression models were used: crude and adjusted. The adjusted model included height, weight, joint pain, alcohol consumption, and smoking status, and a history of chronic diseases, including hypertension, stroke, heart disease, diabetes, dyslipidemia, renal disease, prostate disease, cancer, osteoporosis, other musculoskeletal disorders, Parkinson's disease, dietary intake of protein, fat, and carbohydrate, number of medications, and engagement in sports/exercise and walking. Both ECW and ICW were simultaneously entered to account for their correlation in the thigh (*r* = 0.687; *p* < 0.001; similar for the arm) and to isolate their independent associations with muscle strength. Additionally, these regression analyses were performed to assess the associations between thigh ECW and ICW and measures of lower extremity physical performance using the same models. The variance inflation factor (VIF) was calculated to assess multicollinearity among the input variables. The maximum VIF was 2.70, indicating that multicollinearity was not a major concern in this analysis. Furthermore, multiple linear regression analyses examined the relationships between the ECW/ICW ratio and upper and lower extremity muscle strength and physical performance, using the same crude and adjusted models without ECW or ICW. All analyses were conducted as hypothesis‐driven analyses based on the a priori study aims, and all analyzed outcomes were treated as primary outcomes in this study.

## 3 RESULTS

Table 1 presents the characteristics of the study participants.

**TABLE 1:** Characteristics of the participants.

Variable	Male					Female					Total
	65–69	70–74	75–79	80+	Total	65–69	70–74	75–79	80+	Total	
*n*	173	154	114	94	535	199	189	123	56	567	1102
Age, years, Mean (SD)	67.1 (1.4)	71.9 (1.5)	76.8 (1.4)	82.8 (2.6)	73.3 (5.9)	67.3 (1.3)	71.9 (1.4)	76.9 (1.4)	82.2 (2.2)	72.4 (5.1)	72.8 (5.5)
Height, cm, Mean (SD)	165.7 (5.8)	163.1 (5.3)	161.9 (5.4)	161.3 (6.4)	163.4 (5.9)	152.7 (4.7)	150.4 (5.1)	148.4 (5.3)	147.3 (4.3)	150.5 (5.3)	156.7 (8.5)
Weight, kg, Mean (SD)	64.1 (8.6)	62.1 (8.6)	61.5 (8.2)	58.7 (8.3)	62.0 (8.7)	52.4 (7.6)	51.5 (7.8)	50.7 (8.0)	49.4 (8.6)	51.4 (7.9)	56.6 (9.8)
BMI, kg/m², Mean (SD)	23.3 (2.7)	23.3 (3.0)	23.4 (2.7)	22.5 (2.7)	23.2 (2.8)	22.5 (3.0)	22.8 (3.3)	23.0 (3.5)	22.7 (3.3)	22.7 (3.2)	22.9 (3.0)
Protein intake, g, Mean (SD)	59.1 (12.2)	58.8 (15.7)	64.8 (17.1)	66.1 (17.3)	61.5 (15.6)	54.3 (11.8)	57.7 (15.8)	55.8 (15.3)	57.7 (17.4)	56.1 (14.6)	58.7 (15.3)
Fat intake, g, Mean (SD)	43.9 (11.0)	42.4 (11.3)	45.3 (12.3)	46.8 (13.5)	44.3 (11.9)	42.9 (10.9)	45.2 (13.5)	44.4 (12.6)	51.4 (22.6)	44.8 (13.9)	44.6 (12.9)
Carbohydrate intake, g, Mean (SD)	275.0 (83.8)	277.7 (107.8)	288.1 (75.0)	287.0 (73.8)	280.7 (88.1)	216.8 (57.3)	224.7 (65.5)	212.2 (64.8)	216.9 (72.7)	218.5 (63.4)	248.7 (82.4)
Number of medications, Mean (SD)	3.8 (1.9)	3.6 (1.7)	3.7 (1.7)	3.6 (1.6)	3.7 (1.8)	3.5 (2.0)	3.2 (1.8)	3.9 (1.6)	3.6 (1.7)	3.5 (1.9)	3.6 (1.8)
Sports/exercise, *n* (%)	79 (45.7)	62 (40.3)	46 (40.4)	41 (43.6)	228 (42.6)	97 (48.7)	80 (42.3)	59 (48.0)	18 (32.1)	254 (44.8)	482 (43.7)
Walking habit, *n* (%)	95 (54.9)	89 (57.8)	59 (51.8)	56 (59.6)	299 (55.9)	96 (48.2)	94 (49.7)	52 (42.3)	20 (35.7)	262 (46.2)	561 (50.9)
Joint pain, *n* (%)	50 (28.9)	48 (31.2)	46 (40.4)	35 (37.2)	179 (33.5)	59 (29.6)	82 (43.4)	65 (52.8)	28 (50.0)	234 (41.3)	413 (37.5)
Alcohol consumption											
Almost daily	98 (56.6)	87 (56.5)	54 (47.4)	36 (38.3)	275 (51.4)	17 (8.5)	13 (6.9)	4 (3.3)	1 (1.8)	35 (6.2)	310 (28.1)
Sometimes	24 (13.9)	27 (17.5)	24 (21.1)	15 (16.0)	90 (16.8)	38 (19.1)	27 (14.3)	20 (16.3)	4 (7.1)	89 (15.7)	179 (16.2)
Rarely	31 (17.9)	23 (14.9)	19 (16.7)	21 (22.3)	94 (17.6)	66 (33.2)	52 (27.5)	36 (29.3)	20 (35.7)	174 (30.7)	268 (24.3)
Never	20 (11.6)	17 (11.0)	17 (14.9)	22 (23.4)	76 (14.2)	78 (39.2)	97 (51.3)	63 (51.2)	31 (55.4)	269 (47.4)	345 (31.3)
Smoking status											
Almost daily	30 (17.3)	18 (11.7)	9 (7.9)	8 (8.5)	65 (12.1)	2 (1.0)	6 (3.2)	2 (1.6)	0 (0.0)	10 (1.8)	75 (6.8)
Sometimes	3 (1.7)	2 (1.3)	3 (2.6)	0 (0.0)	8 (1.5)	1 (0.5)	2 (1.1)	0 (0.0)	0 (0.0)	3 (0.5)	11 (1.0)
Quit smoking	108 (62.4)	100 (64.9)	67 (58.8)	57 (60.6)	332 (62.1)	25 (12.6)	6 (3.2)	6 (4.9)	0 (0.0)	37 (6.5)	369 (33.5)
Never smoked	32 (18.5)	34 (22.1)	35 (30.7)	29 (30.9)	130 (24.3)	171 (85.9)	175 (92.6)	115 (93.5)	56 (100.0)	517 (91.2)	647 (58.7)
Hypertension, *n* (%)	56 (32.4)	63 (40.9)	60 (52.6)	38 (40.4)	217 (40.6)	67 (33.7)	82 (43.4)	62 (50.4)	30 (53.6)	241 (42.5)	458 (41.6)
Stroke, *n* (%)	5 (2.9)	5 (3.2)	6 (5.3)	6 (6.4)	22 (4.1)	1 (0.5)	1 (0.5)	4 (3.3)	1 (1.8)	7 (1.2)	29 (2.6)
Heart disease, *n* (%)	22 (12.7)	22 (14.3)	16 (14.0)	18 (19.1)	78 (14.6)	9 (4.5)	10 (5.3)	12 (9.8)	6 (10.7)	37 (6.5)	115 (10.4)
Diabetes, *n* (%)	19 (11.0)	20 (13.0)	12 (10.5)	10 (10.6)	61 (11.4)	13 (6.5)	12 (6.3)	12 (9.8)	3 (5.4)	40 (7.1)	101 (9.2)
Dyslipidemia, *n* (%)	19 (11.0)	15 (9.7)	6 (5.3)	5 (5.3)	45 (8.4)	37 (18.6)	35 (18.5)	11 (8.9)	6 (10.7)	89 (15.7)	134 (12.2)
Renal/Prostate disease, *n* (%)	17 (9.8)	17 (11.0)	19 (16.7)	19 (20.2)	72 (13.5)	0 (0.0)	1 (0.5)	1 (0.8)	1 (1.8)	3 (0.5)	75 (6.8)
Musculoskeletal disease, *n* (%)	7 (4.0)	6 (3.9)	9 (7.9)	7 (7.4)	29 (5.4)	31 (15.6)	39 (20.6)	27 (22.0)	14 (25.0)	111 (19.6)	140 (12.7)
Cancer, *n* (%)	4 (2.3)	9 (5.8)	9 (7.9)	6 (6.4)	28 (5.2)	3 (1.5)	7 (3.7)	6 (4.9)	1 (1.8)	17 (3.0)	45 (4.1)
Knee extension strength, kg, Mean (SD)	39.8 (10.3)	34.4 (9.9)	29.3 (9.0)	26.3 (7.8)	33.6 (10.8)	23.3 (6.3)	21.0 (6.2)	18.5 (5.8)	15.4 (4.6)	20.8 (6.5)	27.1 (10.9)
Grip strength, kg, Mean (SD)	37.3 (5.5)	33.9 (5.1)	31.3 (5.5)	28.9 (5.6)	33.6 (6.2)	23.1 (3.8)	21.8 (3.5)	20.3 (3.3)	18.0 (4.1)	21.6 (3.9)	27.4 (7.9)
Vertical jump index, cm×kg, Mean (SD)	1979.8 (559.4)	1604.8 (456.3)	1312.8 (478.7)	1021.5 (368.8)	1570.4 (596.4)	1116.4 (296.9)	969.2 (284.6)	759.2 (262.2)	615.8 (213.9)	949.1 (324.1)	1261.9 (572.4)
TUG, second, Mean (SD)	6.4 (1.2)	7.2 (2.4)	8.1 (2.7)	8.5 (2.2)	7.4 (2.2)	6.8 (1.2)	7.4 (1.9)	8.0 (1.6)	9.3 (2.0)	7.5 (1.8)	7.4 (2.0)
Six‐meter maximum walk, second, Mean (SD)	3.0 (0.4)	3.3 (0.5)	3.5 (0.7)	3.8 (0.8)	3.3 (0.7)	3.2 (0.4)	3.4 (0.5)	3.7 (0.8)	4.1 (0.7)	3.5 (0.6)	3.4 (0.7)
Chair stand, second, Mean (SD)	8.0 (2.2)	8.4 (2.6)	9.1 (3.3)	10.7 (4.5)	8.8 (3.2)	8.0 (2.3)	8.5 (2.5)	8.9 (2.6)	11.4 (4.0)	8.6 (2.8)	8.7 (3.0)
One leg standing, second, Mean (SD)	58.8 (41.3)	44.6 (39.4)	32.1 (38.2)	17.5 (27.6)	41.9 (40.7)	62.1 (43.7)	44.6 (40.8)	22.2 (22.7)	13.0 (19.2)	43.2 (41.1)	42.5 (40.9)
Thigh ECW, L, Mean (SD)	1686.0 (263.8)	1656.9 (263.7)	1691.0 (300.3)	1692.1 (264.2)	1679.8 (271.7)	1293.1 (220.8)	1274.5 (215.2)	1290.9 (250.4)	1334.0 (328.0)	1290.4 (238.1)	1479.4 (320.7)
Thigh ICW, L, Mean (SD)	4353.1 (912.5)	3979.3 (894.0)	3727.7 (896.3)	3289.0 (815.1)	3925.3 (960.5)	2746.2 (598.1)	2588.5 (539.3)	2395.5 (561.8)	2165.6 (592.2)	2560.2 (598.0)	3222.9 (1047.5)
Thigh TBW, L, Mean (SD)	6039.1 (1104.4)	5636.2 (1081.6)	5418.7 (1122.3)	4981.1 (1034.3)	5605.0 (1148.1)	4039.2 (777.4)	3863.0 (698.6)	3686.4 (771.6)	3499.6 (883.8)	3850.6 (780.0)	4702.4 (1312.1)
Arm ECW, L, Mean (SD)	934.4 (125.9)	922.2 (127.3)	910.1 (127.7)	900.4 (126.4)	919.7 (127.0)	669.5 (102.4)	661.4 (88.6)	678.4 (110.5)	684.3 (124.3)	670.2 (102.3)	791.3 (169.6)
Arm ICW, L, Mean (SD)	2180.5 (408.4)	2031.7 (401.2)	1907.3 (370.6)	1761.8 (374.5)	2005.9 (419.5)	1254.2 (281.4)	1194.4 (238.7)	1185.9 (255.3)	1130.4 (246.4)	1207.2 (261.0)	1595.0 (529.0)
Arm TBW, L, Mean (SD)	3114.9 (501.8)	2953.9 (499.5)	2817.4 (467.3)	2662.2 (475.6)	2925.6 (514.5)	1923.7 (369.7)	1855.8 (310.4)	1864.3 (345.2)	1814.7 (350.2)	1877.4 (344.6)	2386.3 (681.3)

*Note*: The number of variables varied owing to missing or outlier cases.

Abbreviations: BMI, body mass index; ECW, extracellular water; ICW, intracellular water; SD, standard deviation; TBW, total body water.

**FIGURE 1:**
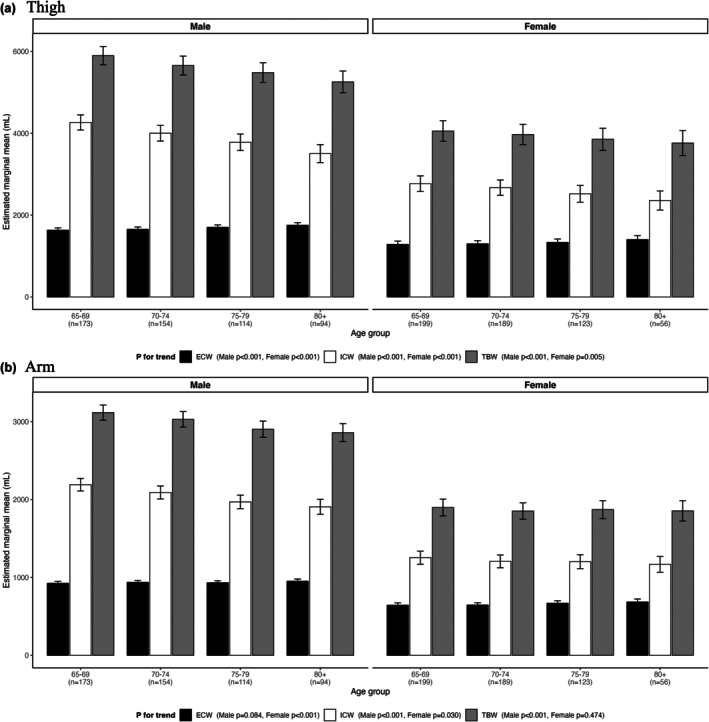
The difference in (a) thigh and (b) arm ECW, ICW, and TBW (adjusted estimated marginal means and 95% confidence interval) among age groups (65–69, 70–74, 75–79, and ≥80 years), adjusted for height, weight, joint pain, status of alcohol consumption and smoking, and history of chronic diseases, including hypertension, stroke, heart disease, diabetes, dyslipidemia, renal disease, prostate disease, cancer, osteoporosis, and other musculoskeletal disorders, Parkinson's disease, dietary intake of protein, fat, and carbohydrate, number of medications, and engagement in sports/exercise and walking. ECW, extracellular water; ICW, intracellular water; TBW, total body water.

Figure 1 displays the estimated marginal means of ECW, ICW, and TBW in the thighs and arms across the four age categories (65–69, 70–74, 75–79, and ≥80 years) obtained from ANCOVA. The corresponding adjusted means and 95% confidence intervals for thigh and arm ECW, ICW, and TBW across age groups, stratified by sex, are presented in Table 2. In the thigh, older age groups exhibited significantly higher ECW and significantly lower ICW and TBW; in the arm, older age groups had significantly lower ICW, whereas ECW and TBW demonstrated similar tendencies, with sex‐specific differences in statistical significance (ECW significant in females only; TBW significant in males only). Specifically, comparing the youngest (65–69 years) with the oldest (≥80 years) group for thigh measurements, ICW was 17.9% and 14.8% lower, TBW was 10.9% and 7.3% lower, and ECW was 7.3% and 8.9% higher in men and women, respectively.

**TABLE 2:** Adjusted means and 95% confidence intervals for thigh and arm ECW, ICW, and TBW across age groups by sex.

			65–69 years		70–74 years		75–79 years		80+ years	
			Adjusted mean	95%CI	Adjusted mean	95% CI	Adjusted mean	95% CI	Adjusted mean	95% CI
Thigh	Male	ECW	1634	(1579–1688)	1655	(1599–1711)	1703	(1644–1762)	1753	(1689–1818)
		ICW	4267	(4082–4452)	4003	(3812–4194)	3784	(3584–3984)	3505	(3286–3725)
		TBW	5901	(5677–6124)	5658	(5427–5889)	5487	(5246–5729)	5259	(4993–5524)
	Female	ECW	1288	(1210–1366)	1301	(1224–1378)	1335	(1251–1418)	1403	(1308–1499)
		ICW	2769	(2577–2961)	2672	(2483–2860)	2521	(2317–2726)	2359	(2126–2593)
		TBW	4057	(3804–4310)	3973	(3724–4222)	3856	(3586–4125)	3762	(3454–4071)
Arm	Male	ECW	926	(903–949)	939	(915–963)	933	(908–958)	953	(925–980)
		ICW	2192	(2111–2272)	2091	(2008–2174)	1970	(1883–2057)	1907	(1812–2003)
		TBW	3118	(3021–3215)	3031	(2931–3131)	2903	(2799–3008)	2860	(2745–2975)
	Female	ECW	644	(614–675)	646	(617–676)	669	(637–702)	686	(649–723)
		ICW	1254	(1170–1338)	1207	(1124–1289)	1203	(1113–1292)	1170	(1068–1272)
		TBW	1898	(1791–2006)	1853	(1747–1959)	1872	(1757–1987)	1856	(1725–1987)

*Note*: Values are adjusted means and 95% confidence intervals derived from ANCOVA. Adjusted for height, weight, joint pain, alcohol consumption, smoking status, history of chronic diseases (hypertension, stroke, heart disease, diabetes, dyslipidemia, renal disease, prostate disease, cancer, osteoporosis, other musculoskeletal disorders, and Parkinson's disease), dietary intake of protein, fat, and carbohydrate, number of medications, and engagement in sports/exercise and walking.

Abbreviations: CI, confidence interval; ECW, extracellular water; ICW, intracellular water; TBW, total body water.

Table 3 presents the associations between thigh and arm ECW and ICW and muscle strength. In the crude models, both ECW and ICW remained positively associated with strength. In the adjusted models, thigh and arm ICW were positively associated with the corresponding strength measures, whereas ECW was negatively associated.

**TABLE 3:** Association between thigh and arm ECW and ICW and lower and upper extremity muscle strengths.

			Crude model			Adjusted model		
			*β*	95% CI	*p* Value	*β*	95% CI	*p* Value
Knee extension strength	Thigh ECW	Male	0.177	0.096 to 0.258	<0.001	−0.143	−0.245 to −0.041	0.006
		Female	0.095	0.015 to 0.175	0.020	−0.237	−0.348 to −0.127	<0.001
	Thigh ICW	Male	0.468	0.395 to 0.541	<0.001	0.338	0.231 to 0.446	<0.001
		Female	0.348	0.273 to 0.424	<0.001	0.344	0.230 to 0.458	<0.001
Grip strength	Arm ECW	Male	0.396	0.322 to 0.471	<0.001	−0.120	−0.216 to −0.025	0.014
		Female	0.304	0.229 to 0.379	<0.001	−0.199	−0.305 to −0.093	<0.001
	Arm ICW	Male	0.576	0.510 to 0.642	<0.001	0.428	0.337 to 0.518	<0.001
		Female	0.459	0.390 to 0.529	<0.001	0.482	0.385 to 0.579	<0.001

*Note*: Adjusted model: height, weight, joint pain, status of alcohol consumption and smoking, and history of chronic diseases, including hypertension, stroke, heart disease, diabetes, dyslipidemia, renal disease, prostate disease, cancer, osteoporosis, and other musculoskeletal disorders and Parkinson's disease, dietary intake of protein, fat, and carbohydrate, number of medications, and engagement in sports/exercise and walking, and ICW or ECW (i.e., if ICW was entered as an explanatory variable, ECW was entered as an adjusted variable).

Abbreviations: β, standardized regression coefficient; CI, confidence interval; ECW, extracellular water; ICW, intracellular water.

Table 4 shows the associations of thigh ECW and ICW with physical performance. In the adjusted models, higher thigh ICW was consistently associated with better performance, whereas higher thigh ECW was associated with poorer performance across all lower‐limb function measures, including mobility, power, and balance.

**TABLE 4:** Association between thigh ECW and ICW and lower extremity physical performance.

			Crude model		*p* Value	Adjusted model		*p* Value
			*β*	95% CI		*β*	95% CI	
Vertical jump index	Thigh ECW	Male	0.190	0.107 to 0.273	<0.001	−0.248	−0.336 to −0.160	<0.001
		Female	0.129	0.046 to 0.213	0.002	−0.310	−0.406 to −0.215	<0.001
	Thigh ICW	Male	0.561	0.491 to 0.631	<0.001	0.441	0.349 to 0.533	<0.001
		Female	0.435	0.360 to 0.511	<0.001	0.380	0.281 to 0.478	<0.001
Chair stand	Thigh ECW	Male	−0.005	−0.088 to 0.078	0.903	0.149	0.028 to 0.271	0.016
		Female	0.063	−0.017 to 0.144	0.123	0.170	0.049 to 0.292	0.006
	Thigh ICW	Male	−0.229	−0.310 to −0.148	<0.001	−0.244	−0.373 to −0.116	<0.001
		Female	−0.149	−0.229 to −0.069	<0.001	−0.230	−0.355 to −0.105	<0.001
Six‐meter maximum walk	Thigh ECW	Male	0.021	−0.061 to 0.103	0.620	0.255	0.144 to 0.365	<0.001
		Female	0.074	−0.006 to 0.154	0.071	0.261	0.155 to 0.368	<0.001
	Thigh ICW	Male	−0.291	−0.369 to −0.212	<0.001	−0.331	−0.447 to −0.215	<0.001
		Female	−0.191	−0.270 to −0.112	<0.001	−0.196	−0.305 to −0.087	<0.001
Timed up‐and‐go	Thigh ECW	Male	−0.027	−0.110 to 0.055	0.517	0.175	0.062 to 0.288	0.002
		Female	0.032	−0.048 to 0.112	0.430	0.202	0.099 to 0.305	<0.001
	Thigh ICW	Male	−0.297	−0.376 to −0.218	<0.001	−0.337	−0.456 to −0.218	<0.001
		Female	−0.239	−0.316 to −0.162	<0.001	−0.232	−0.338 to −0.126	<0.001
Single leg balance with eyes open	Thigh ECW	Male	−0.030	−0.112 to 0.053	0.481	−0.155	−0.271 to −0.039	0.009
		Female	−0.064	−0.144 to 0.016	0.117	−0.122	−0.233 to −0.012	0.029
	Thigh ICW	Male	0.254	0.174 to 0.333	<0.001	0.253	0.131 to 0.375	<0.001
		Female	0.154	0.074 to 0.233	<0.001	0.155	0.042 to 0.269	0.008

*Note*: Adjusted model: height, weight, joint pain, status of alcohol consumption and smoking, and history of chronic diseases, including hypertension, stroke, heart disease, diabetes, dyslipidemia, renal disease, prostate disease, cancer, osteoporosis, and other musculoskeletal disorders and Parkinson's disease, dietary intake of protein, fat, and carbohydrate, number of medications, and engagement in sports/exercise and walking, and ICW or ECW (i.e., if ICW was entered as an explanatory variable, ECW was entered as an adjusted variable).

Abbreviations: β, standardized regression coefficient; CI, confidence interval; ECW, extracellular water; ICW, intracellular water.

Table 5 presents the associations between the ECW/ICW ratio and muscle strength and physical performance. In both crude and adjusted models, a higher thigh ECW/ICW ratio was associated with poorer lower‐limb physical performance, whereas a higher arm ECW/ICW ratio was associated with lower handgrip strength.

**TABLE 5:** Association between thigh and arm ECW/ICW ratio and muscle strength and physical performance.

		Crude model			Adjusted model		
		*β*	95% CI	*p* Value	*β*	95% CI	*p* Value
Arm ECW/ICW							
Grip strength	Male	−0.426	−0.498 to −0.353	<0.001	−0.283	−0.350 to −0.217	<0.001
	Female	−0.370	−0.443 to −0.297	<0.001	−0.323	−0.388 to −0.259	<0.001
Thigh ECW/ICW							
Knee extension strength	Male	−0.459	−0.531 to −0.387	<0.001	−0.272	−0.356 to −0.189	<0.001
	Female	−0.383	−0.457 to −0.309	<0.001	−0.237	−0.319 to −0.155	<0.001
Vertical jump index	Male	−0.550	−0.619 to −0.480	<0.001	−0.326	−0.399 to −0.253	<0.001
	Female	−0.463	−0.538 to −0.389	<0.001	−0.258	−0.329 to −0.186	<0.001
Chair stand	Male	0.297	0.219 to 0.375	<0.001	0.219	0.119 to 0.318	<0.001
	Female	0.276	0.198 to 0.354	<0.001	0.161	0.071 to 0.250	<0.001
Six‐meter maximum walk	Male	0.366	0.290 to 0.441	<0.001	0.253	0.163 to 0.344	<0.001
	Female	0.338	0.263 to 0.414	<0.001	0.160	0.081 to 0.239	<0.001
Timed up‐and‐go	Male	0.358	0.282 to 0.434	<0.001	0.278	0.186 to 0.371	<0.001
	Female	0.360	0.285 to 0.435	<0.001	0.186	0.107 to 0.264	<0.001
Single leg balance with eyes open	Male	−0.349	−0.425 to −0.273	<0.001	−0.208	−0.303 to −0.114	<0.001
	Female	−0.279	−0.356 to −0.202	<0.001	−0.114	−0.196 to −0.032	0.006

*Note*: Adjusted model: height, weight, joint pain, alcohol consumption, smoking status, history of chronic diseases (hypertension, stroke, heart disease, diabetes, dyslipidemia, renal disease, prostate disease, cancer, osteoporosis, other musculoskeletal disorders, and Parkinson's disease), dietary intake of protein, fat, and carbohydrate, number of medications, and engagement in sports/exercise and walking.

Abbreviations: CI, confidence interval; ECW, extracellular water; ICW, intracellular water.

## 4 DISCUSSION

This study examined age‐related differences in ECW and ICW in the upper and lower limbs, as well as their associations with muscle strength and physical performance in over 1100 community‐dwelling older adults. After adjustment for various covariates, ECW was negatively associated with muscle strength in the corresponding limbs (arms and thighs), whereas ICW was positively associated. Thigh ECW was also negatively related to lower‐limb mobility, explosive power, balance, and chair standing. The standardized regression coefficients for thigh ECW and ICW ranged from |β| = 0.12–0.31 and 0.16–0.48, respectively. Arm ECW and ICW were also significantly associated with grip strength (|β| = 0.12 and 0.43 in males; 0.20 and 0.48 in females). These effect sizes are comparable to established predictors of functional decline. Additionally, age‐related trend analysis with ANCOVA revealed that in the thigh, older age groups had significantly higher ECW and significantly lower ICW and TBW. In the arm, older age groups showed significantly lower ICW, whereas ECW and TBW followed similar trends, with sex‐specific differences in statistical significance (ECW significant in females only; TBW significant in males only). Therefore, composite indices such as TBW or conventional muscle mass may underestimate muscle quality deterioration, supporting the thesis that ECW should be considered an independent correlate of functional decline.

ECW was negatively associated with muscle strength and physical performance. Although ECW has been traditionally considered a non‐contractile, nonfunctional component that does not directly contribute to force generation (Lorenzo et al., 2019; Yamada, 2018; Yamada et al., 2010), our results suggest that it is associated with poorer muscle function. This finding aligns with the results of reports from animal models demonstrating that excess ECM and IMAT impair force transmission and reduce muscle strength (Biltz et al., 2020; Zhang & Gao, 2014). Therefore, our study provides novel evidence from a large human cohort that ECW may serve as a marker of extracellular fluid redistribution accompaying increases in nonfunctional structural components.

As hypothesized, ICW was positively associated with muscle strength and physical performance; it reflects the volume of water within muscle cells, which is considered a functional muscle mass indicator (Lorenzo et al., 2019; Yamada, 2018; Yamada et al., 2010). An ICW decline may have adverse consequences beyond solely reducing functional mass. First, reduced ICW may induce hyperosmotic stress and cellular dehydration, leading to a relative increase in intracellular macromolecular concentration. This imbalance in the cellular environment may compromise the stability and function of intracellular protein structures (Lorenzo et al., 2019), potentially affecting cell function and metabolism. Additionally, increased protein osmotic pressure may alter the chemical potential of contractile proteins, increasing muscle structural stiffness and the viscosity between actin and myosin filaments (Grazi, 2008). Furthermore, disruption of the ordered structure of the exclusion zone water layer may eliminate its lubricating effect on protein surfaces, potentially reducing the lubricity of the actin–myosin cross‐bridges. Notably, these changes may inhibit cross‐bridge formation and dissociation, thereby potentially reducing muscle contraction power output and velocity (Lorenzo et al., 2019). Thus, the association between lower ICW and reduced muscle strength and physical function may reflect both decreased functional muscle mass and conditions less favorable to muscle contraction and metabolism.

Additionally, chronic effects must be considered. Water is an essential nutrient vital for metabolic, transport, structural, and thermoregulatory functions (Lorenzo et al., 2019). Its distribution is closely associated with myocyte health and function. Chronic ICW reductions may impair cellular homeostasis and the anabolic environment. Conversely, chronic ECW excess may induce hyperosmolarity, potentially leading to oxidative stress, cellular dysfunction, apoptosis, and cell death; moreover, it may suppress muscle satellite cell activation and differentiation, thereby potentially impairing repair of muscle damage (Brocker et al., 2012). This may contribute to progressive muscle atrophy and fibrosis, potentially leading to a decline in muscle strength and physical function and an increased disability risk. Conversely, increased ICW and cell volume may activate cellular metabolism, intracellular signaling, and mitochondrial function, promoting anabolism (Häussinger, 1996; Lang et al., 1998). Furthermore, ECW may be influenced by broader physiological processes, including inflammation, vascular function, and disease‐related conditions (Asano, Miyachi, et al., 2025; Lorenzo et al., 2019), which may also contribute to the observed associations with muscle function. Alternative explanations should also be considered. For example, reduced physical activity or functional decline itself may contribute to altered fluid distribution, and the observed associations may partly reflect broader systemic processes rather than muscle‐specific changes alone. Therefore, the balance between ECW and ICW may influence biological functions through multiple pathways, a mechanism that warrants further longitudinal investigation.

Although age‐related decreases in ICW are consistently reported, findings on age‐related changes in ECW have been mixed. Some studies and populations have reported stability (Silva et al., 2005, 2007; Yamada et al., 2010, 2014), whereas others have found an increase (Ohashi et al., 2018; Silva et al., 2005, 2007). These discrepancies may stem from differences in key study characteristics, including participant demographics (race and sex), the control of measurement conditions, measurement methodology and accuracy, use of whole‐body versus segmental analysis, and the extent of covariate adjustment. The gold standard for assessing whole‐body water distribution is a combination of deuterium dilution, bromide dilution, and total body potassium measurements. Nonetheless, these methods are costly and require extensive equipment. Furthermore, they cannot assess segmental fluid distribution, for which no gold standard exists (Yamada, 2018). Water distribution estimates from BIS are comparable to those from these gold standard methods (Segal et al., 1991). Our study addressed the limitations of prior techniques by using high‐precision segmental BIS. This method uses Cole‐Cole plot analysis across >200 frequencies, combined with segmental length measurements (Lorenzo et al., 1997). Using this approach in a large cohort and adjusting for many covariates, we found that older age groups had significantly higher ECW and lower ICW and TBW in the thigh. In the arm, similar tendencies were observed, though with sex‐specific differences in statistical significance.

Traditional methods that use TBW as a proxy for muscle mass (e.g., DXA, BIA, and imaging methods) may underestimate the actual extent of muscle atrophy. Specifically, in the thigh, ICW was 17.9% lower in male and 14.8% lower in female, whereas ECW was 7.3% higher in male and 8.9% higher in female when comparing the oldest group with the youngest group. Consequently, TBW decreased by only 10.9% in male and 7.3% in female. Because ECW represents the extracellular fluid compartment rather than functional muscle mass, it may partially mask the true extent of muscle loss. These results emphasize the need for measurement techniques that distinguish functional from nonfunctional muscle components in future assessments.

The ECW/TBW and ECW/ICW ratios are gaining attention as clinically accessible biomarkers because they can be easily, quickly, and noninvasively estimated for the whole body and each segment using BIA devices, without requiring specialized staff or large equipment (Dimassi et al., 2024; Jaffrin & Morel, 2008; Khalil et al., 2014; Sergi et al., 2016). The whole‐body ECW/TBW and ECW/ICW ratios are associated with frailty, sarcopenia, long‐term care needs, and mortality (Hioka et al., 2021; Iwasaka et al., 2023, 2024; Tanaka et al., 2020). Additionally, the lower limb ratio is more strongly associated with lower limb physical function and the incidence of functional disability (Asano et al., 2023; Asano, Tsunoda, et al., 2025). In this context, ECW/ICW ratio analysis in this study serves as a complementary extension of the separate ECW and ICW analyses, and its originality lies in evaluating this composite marker in a larger sample, with broader covariate adjustment, using high‐precision BIS, and in relation to region‐matched muscle strength and physical performance measures in both the arm and thigh.

This study suggests that ECW and ICW are important correlates of the age‐related decline in muscle strength and physical function in older adults. Previously, using the ECW/ICW ratio as a single biomarker lacked an understanding of the distinct physiological implications of excess ECW accumulation versus ICW depletion. Our findings promote a more precise physiological understanding by demonstrating the importance of assessing the dynamics of both ECW and ICW when interpreting the ratio, thereby facilitating its implementation in clinical settings. Future studies should focus on interventions specifically designed to enhance ICW and reduce ECW, such as targeted strength training and nutritional or pharmacological strategies, and should develop and rigorously evaluate their effectiveness. Additionally, cross‐validation of BIS with other methods for assessing functional muscle mass and the development of international diagnostic protocols using these techniques are necessary to advance their practical application.

This study has several strengths, including its large sample size (over 1100 community‐dwelling older adults aged 65–90 years), sex‐specific analyses of ECW and ICW measured by BIS, and adjustment for a wide range of covariates. Nonetheless, this study has some limitations. First, the cross‐sectional design precludes establishing causality among fluid distribution, aging, and functional decline. It is unclear whether chronic increases in ECW or decreases in ICW cause muscle dysfunction or if inactivity resulting from poor muscle function leads to fluid imbalances. Additionally, as we did not follow up with the same individuals over time, the observed age‐related changes may not accurately reflect individual aging trajectories. Second, the study participants were volunteers and may therefore have been healthier and more health‐conscious than the general older population. Given the participation rate and exclusion criteria, sampling bias may also have been present. As a result, the observed associations with functional decline may have been underestimated, particularly because individuals with frailty or those who died early were not included in the analysis. The generalizability of these findings may therefore be limited, especially for frailer older adults. In addition, because this study included only older adults, caution is warranted when extrapolating these findings to other age groups, and future studies including younger adults will be necessary to better contextualize these findings across the adult lifespan. Although the study was conducted across 10 regions to minimize this bias, caution is warranted when generalizing the results to the broader population. Third, ECW and ICW estimates may be biased because they assume constant ion concentrations and fluid resistivity coefficients. Nevertheless, we estimated resistance values (Re and Ri) with relatively high accuracy using data from 256 frequencies and incorporating segmental length. Additionally, because BIS assessments were conducted under field conditions, food and beverage intake, medication use, and time of day were not fully standardized. Although participants rested in a supine position before measurement, residual variability related to hydration status cannot be fully excluded. Fourth, this study was conducted in a single region with a unique population. Because ECW and ICW distributions can vary by ethnicity and demographics, additional studies in other populations are warranted to confirm these findings. Fifth, although this study used standardized protocols for BIS‐derived fluid estimation and for muscle strength and physical performance assessments, these measurements may still be subject to intra‐individual variability and measurement error. Furthermore, although we adjusted for a wide range of covariates, residual confounding cannot be fully excluded, including the potential influence of disease burden and subclinical pathology. Therefore, the observed associations should be interpreted with caution.

## 5 CONCLUSION

In this large cohort of community‐dwelling older adults, we applied segmental bioelectrical impedance spectroscopy to estimate ECW and ICW in the upper and lower limbs. Limb ICW may decrease with advancing age, whereas ECW may increase with aging, suggesting that loss of intracellular “functional muscle” and expansion of extracellular fluid volume within the non‐contractile compartment may occur in parallel. Importantly, higher limb ECW was independently and negatively associated with muscle strength and physical performance, even after accounting for conventional covariates and ICW. Thus, ECW may not merely be a neutral, non‐contractile compartment, but may indicate a fluid‐related tissue compartment that could interfere with effective force generation and contribute to lower muscle function. These results provide a water‐compartment‐based perspective on muscle quality and indicate that, beyond “functional muscle mass” and the relative loss of contractile tissue, quantifying non‐contractile elements, such as ECW itself, may be essential for understanding age‐related declines in muscle strength and physical performance.

## AUTHOR CONTRIBUTIONS

**Yujiro Asano:** Conceptualization; data curation; formal analysis; methodology; software; validation; visualization. **Tsukasa Yoshida:** Conceptualization; data curation; funding acquisition; investigation; methodology; software; validation. **Kenji Tsunoda:** Conceptualization; formal analysis; methodology; validation. **Keiichi Yokoyama:** Investigation. **Yuya Watanabe:** Investigation; validation. **Yasuko Yoshinaka:** Investigation. **Tomohiro Okura:** Supervision. **Misaka Kimura:** Conceptualization; investigation; methodology; validation. **Yosuke Yamada:** Conceptualization; data curation; investigation; methodology; supervision.

## FUNDING INFORMATION

This study was supported by the JSPS KAKENHI 23KJ0273 for YA, 24H00683 for YY, and 19K20140, 22H03525, and 24K22286 for TY.

## CONFLICT OF INTEREST STATEMENT

None of the authors have any conflict of interest to disclose.

## ETHICS STATEMENT

We confirm that we have read the Journal's position on issues involved in ethical publication and affirm that this report is consistent with those guidelines.

## CONSENT STATEMENT

All the participants provided informed consent, and this study was approved by the Ethics Committee of the Kyoto Prefectural University of Medicine (No. RBMR‐E‐363), Kyoto University of Advanced Science (No. 20‐1), and National Institute of Health and Nutrition (No. NIHN187‐3 and 25M22) and adhered to the principles outlined in the Declaration of Helsinki.

## Supporting information


Appendix S1.

## Data Availability

The data are not publicly available because of privacy and ethical restrictions. The data supporting the findings of this study are available from the corresponding author upon request.
