# Supplementary material for: Extracellular water may increase with age and be independently and negatively associated with muscle strength and physical function in older adults: A cross‐sectional study
Source: Physiol Rep. 2026 Jun 10;14(11):e70931. doi: 10.14814/phy2.70931 (PMC13250462; doi:10.14814/phy2.70931)
Supplement: Supplementary file 1 — Appendix S1. [file PHY2-14-e70931-s001.docx]

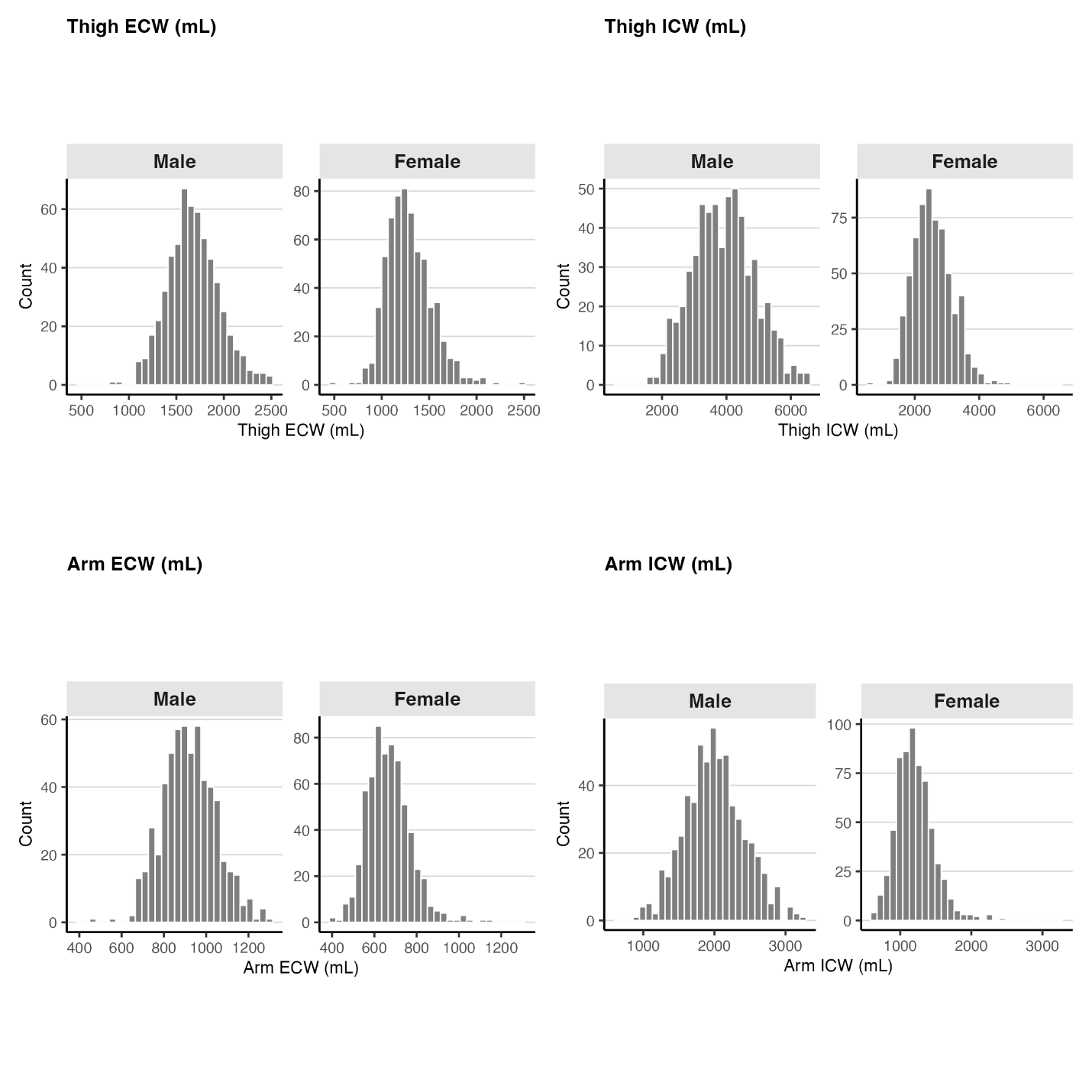
**Supplementary Figure 1. Distribution of segmental extracellular and intracellular water volumes by sex.**

Note. ECW, extracellular water; ICW, intracellular water.


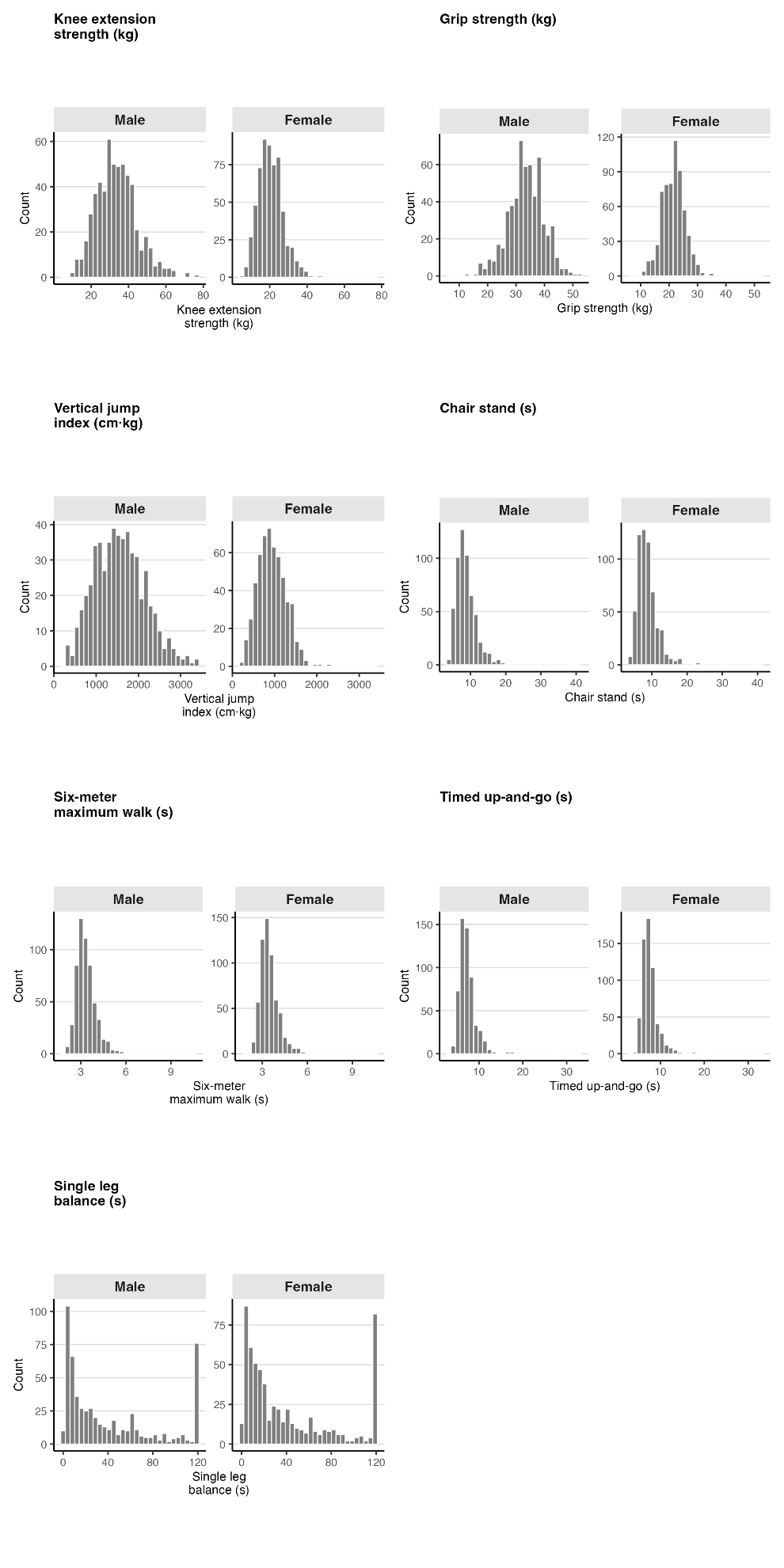


**Supplementary Figure 2. Distribution of physical performance variables by sex.**

Note. Knee extension strength, kg; Grip strength, kg; Vertical jump index, cm×kg (calculated as vertical jump height × body mass); Chair stand, seconds for five repetitions; Six-meter maximum walk, seconds; Timed up-and-go, seconds; Single leg balance with eyes open, seconds (maximum 120 s).

Supplementary Table 1. Association between thigh and arm ECW and ICW and lower and upper extremity muscle strengths with unstandardized regression coefficient.

|  |  |  | Crude model | | | | |  | Adjusted model | | | | |
| --- | --- | --- | --- | --- | --- | --- | --- | --- | --- | --- | --- | --- | --- |
|  |  |  | B | 95% CI | | | p-value |  | B | 95% CI | | | p-value |
| Knee extension strength | Thigh ECW | Male | 0.0070 | 0.0038 | – | 0.010 | < 0.001 |  | -0.0057 | -0.0097 | – | -0.0016 | 0.006 |
|  |  | Female | 0.0026 | 0.00040 | – | 0.0048 | 0.020 |  | -0.0065 | -0.0096 | – | -0.0035 | < 0.001 |
|  | Thigh ICW | Male | 0.0052 | 0.0044 | – | 0.0060 | < 0.001 |  | 0.0038 | 0.0026 | – | 0.0050 | < 0.001 |
|  |  | Female | 0.0037 | 0.0029 | – | 0.0045 | < 0.001 |  | 0.0037 | 0.0024 | – | 0.0049 | < 0.001 |
|  |  |  |  |  |  |  |  |  |  |  |  |  |  |
| Grip strength | Thigh ECW | Male | 0.020 | 0.016 | – | 0.024 | < 0.001 |  | -0.0060 | -0.011 | – | -0.0012 | 0.014 |
|  |  | Female | 0.012 | 0.0090 | – | 0.015 | < 0.001 |  | -0.0078 | -0.012 | – | -0.0037 | < 0.001 |
|  | Thigh ICW | Male | 0.0087 | 0.0077 | – | 0.0097 | < 0.001 |  | 0.0065 | 0.0051 | – | 0.0078 | < 0.001 |
|  |  | Female | 0.0070 | 0.0059 | – | 0.0080 | < 0.001 |  | 0.0073 | 0.0058 | – | 0.0088 | < 0.001 |

Note. B, unstandardized regression coefficient; CI, confidence interval; ECW, extracellular water; ICW, intracellular water. Adjusted model: height, weight, joint pain, status of alcohol consumption and smoking, and history of chronic diseases, including hypertension, stroke, heart disease, diabetes, dyslipidemia, renal disease, prostate disease, cancer, osteoporosis, and other musculoskeletal disorders and Parkinson’s disease, dietary intake of protein, fat, and carbohydrate, number of medications, and engagement in sports/exercise and walking, and ICW or ECW (i.e., if ICW was entered as an explanatory variable, ECW was entered as an adjusted variable).

Supplementary Table 2. Association between thigh ECW and ICW and lower extremity physical performance with unstandardized regression coefficient.

|  |  |  | Crude model | | | | |  | Adjusted model | | | | |
| --- | --- | --- | --- | --- | --- | --- | --- | --- | --- | --- | --- | --- | --- |
|  |  |  | B | 95% CI | | | p-value |  | B | 95% CI | | | p-value |
| Vertical jump index | Thigh ECW | Male | 0.42 | 0.24 | – | 0.60 | < 0.001 |  | -0.55 | -0.74 | – | -0.35 | < 0.001 |
|  |  | Female | 0.18 | 0.064 | – | 0.30 | 0.002 |  | -0.44 | -0.57 | – | -0.30 | < 0.001 |
|  | Thigh ICW | Male | 0.35 | 0.30 | – | 0.39 | < 0.001 |  | 0.27 | 0.22 | – | 0.33 | < 0.001 |
|  |  | Female | 0.24 | 0.20 | – | 0.28 | < 0.001 |  | 0.21 | 0.15 | – | 0.26 | < 0.001 |
|  |  |  |  |  |  |  |  |  |  |  |  |  |  |
| Chair stand | Thigh ECW | Male | -0.000060 | -0.0010 | – | 0.00090 | 0.903 |  | 0.0017 | 0.00032 | – | 0.0031 | 0.016 |
|  |  | Female | 0.00076 | -0.00021 | – | 0.0017 | 0.123 |  | 0.0020 | 0.00058 | – | 0.0035 | 0.006 |
|  | Thigh ICW | Male | -0.00074 | -0.0010 | – | -0.00048 | < 0.001 |  | -0.00079 | -0.0012 | – | -0.00037 | < 0.001 |
|  |  | Female | -0.00070 | -0.0011 | – | -0.00032 | < 0.001 |  | -0.0011 | -0.0017 | – | -0.00049 | < 0.001 |
|  |  |  |  |  |  |  |  |  |  |  |  |  |  |
| Six-meter maximum walk | Thigh ECW | Male | 0.000051 | -0.00015 | – | 0.00025 | 0.620 |  | 0.00062 | 0.00035 | – | 0.00089 | < 0.001 |
|  |  | Female | 0.00020 | -0.000017 | – | 0.00042 | 0.071 |  | 0.00071 | 0.00042 | – | 0.0010 | < 0.001 |
|  | Thigh ICW | Male | -0.00020 | -0.00025 | – | -0.00015 | < 0.001 |  | -0.00023 | -0.00031 | – | -0.00015 | < 0.001 |
|  |  | Female | -0.00020 | -0.00029 | – | -0.00012 | < 0.001 |  | -0.00021 | -0.00033 | – | -0.000093 | < 0.001 |
|  |  |  |  |  |  |  |  |  |  |  |  |  |  |
| Timed up-and-go | Thigh ECW | Male | -0.00022 | -0.00090 | – | 0.00045 | 0.517 |  | 0.0014 | 0.00051 | – | 0.0024 | 0.002 |
|  |  | Female | 0.00028 | -0.00042 | – | 0.00098 | 0.430 |  | 0.0018 | 0.00087 | – | 0.0027 | < 0.001 |
|  | Thigh ICW | Male | -0.00068 | -0.00086 | – | -0.00050 | < 0.001 |  | -0.00077 | -0.0010 | – | -0.00050 | < 0.001 |
|  |  | Female | -0.00082 | -0.0011 | – | -0.00055 | < 0.001 |  | -0.00079 | -0.0012 | – | -0.00043 | < 0.001 |
|  |  |  |  |  |  |  |  |  |  |  |  |  |  |
| Single leg balance with eyes open | Thigh ECW | Male | -0.0045 | -0.017 | – | 0.0080 | 0.481 |  | -0.023 | -0.041 | – | -0.0059 | 0.009 |
|  |  | Female | -0.011 | -0.025 | – | 0.0027 | 0.117 |  | -0.021 | -0.040 | – | -0.0021 | 0.029 |
|  | Thigh ICW | Male | 0.011 | 0.0074 | – | 0.014 | < 0.001 |  | 0.011 | 0.0056 | – | 0.016 | < 0.001 |
|  |  | Female | 0.010 | 0.0050 | – | 0.016 | < 0.001 |  | 0.010 | 0.0028 | – | 0.018 | 0.008 |

Note. B, unstandardized regression coefficient; CI, confidence interval; ECW, extracellular water; ICW, intracellular water

Adjusted model: height, weight, joint pain, status of alcohol consumption and smoking, and history of chronic diseases, including hypertension, stroke, heart disease, diabetes, dyslipidemia, renal disease, prostate disease, cancer, osteoporosis, and other musculoskeletal disorders and Parkinson’s disease, dietary intake of protein, fat, and carbohydrate, number of medications, and engagement in sports/exercise and walking, and ICW or ECW (i.e., if ICW was entered as an explanatory variable, ECW was entered as an adjusted variable).

Supplementary Table 3. Association between thigh and arm ECW/ICW ratio and muscle strength and physical performance with unstandardized regression coefficient.

|  |  | Crude model | | | | |  | Adjusted model | | | | |
| --- | --- | --- | --- | --- | --- | --- | --- | --- | --- | --- | --- | --- |
|  |  | β | 95% CI | | | p-value |  | β | 95% CI | | | p-value |
| Arm ECW/ICW | | | | | | | | | | | | |
| Grip strength | Male | -36 | -42 | – | -30 | < 0.001 |  | -24 | -30 | – | -18 | < 0.001 |
|  | Female | -19 | -23 | – | -15 | < 0.001 |  | -17 | -20 | – | -13 | < 0.001 |
|  |  |  |  |  |  |  |  |  |  |  |  |  |
| Thigh ECW/ICW | | | | | | | | | | | | |
| Knee extension strength | Male | -55 | -64 | – | -46 | < 0.001 |  | -33 | -43 | – | -23 | < 0.001 |
|  | Female | -29 | -34 | – | -23 | < 0.001 |  | -18 | -24 | – | -12 | < 0.001 |
|  |  |  |  |  |  |  |  |  |  |  |  |  |
| Vertical jump index | Male | -3800 | -4200 | – | -3300 | < 0.001 |  | -2200 | -2700 | – | -1700 | < 0.001 |
|  | Female | -1800 | -2100 | – | -1500 | < 0.001 |  | -990 | -1300 | – | -720 | < 0.001 |
|  |  |  |  |  |  |  |  |  |  |  |  |  |
| Chair stand | Male | 10 | 7.5 | – | 13 | < 0.001 |  | 7.5 | 4.1 | – | 11 | < 0.001 |
|  | Female | 9.1 | 6.5 | – | 12 | < 0.001 |  | 5.3 | 2.3 | – | 8.2 | < 0.001 |
|  |  |  |  |  |  |  |  |  |  |  |  |  |
| Six-meter maximum walk | Male | 2.7 | 2.1 | – | 3.2 | < 0.001 |  | 1.8 | 1.2 | – | 2.5 | < 0.001 |
|  | Female | 2.5 | 2.0 | – | 3.1 | < 0.001 |  | 1.2 | 0.60 | – | 1.8 | < 0.001 |
|  |  |  |  |  |  |  |  |  |  |  |  |  |
| Timed up-and-go | Male | 8.9 | 7.0 | – | 11 | < 0.001 |  | 6.9 | 4.6 | – | 9.2 | < 0.001 |
|  | Female | 8.3 | 6.6 | – | 10 | < 0.001 |  | 4.3 | 2.5 | – | 6.1 | < 0.001 |
|  |  |  |  |  |  |  |  |  |  |  |  |  |
| Single leg balance with eyes open | Male | -160 | -190 | – | -120 | < 0.001 |  | -95 | -140 | – | -52 | < 0.001 |
|  | Female | -130 | -170 | – | -95 | < 0.001 |  | -54 | -92 | – | -15 | 0.006 |

Note. CI, confidence interval; ECW, extracellular water; ICW, intracellular water, Adjusted model: height, weight, joint pain, alcohol consumption, smoking status, history of chronic diseases (hypertension, stroke, heart disease, diabetes, dyslipidemia, renal disease, prostate disease, cancer, osteoporosis, other musculoskeletal disorders, and Parkinson’s disease), dietary intake of protein, fat, and carbohydrate, number of medications, and engagement in sports/exercise and walking.
